# Association Between Elevated suPAR, a New Biomarker of Inflammation, and Accelerated Aging

**DOI:** 10.1093/gerona/glaa178

**Published:** 2020-07-16

**Authors:** Line Jee Hartmann Rasmussen, Avshalom Caspi, Antony Ambler, Andrea Danese, Maxwell Elliott, Jesper Eugen-Olsen, Ahmad R Hariri, HonaLee Harrington, Renate Houts, Richie Poulton, Sandhya Ramrakha, Karen Sugden, Benjamin Williams, Terrie E Moffitt

**Affiliations:** 1 Department of Psychology and Neuroscience, Duke University, Durham, North Carolina; 2 Department of Clinical Research, Copenhagen University Hospital Amager and Hvidovre, Hvidovre, Denmark; 3 Social, Genetic, and Developmental Psychiatry Centre, Institute of Psychiatry, Psychology, and Neuroscience, King’s College London, UK; 4 Department of Psychology, University of Otago, Dunedin, New Zealand; 5 Department of Child and Adolescent Psychiatry, Institute of Psychiatry, Psychology, and Neuroscience, King’s College London, UK; 6 National and Specialist Child and Adolescent Mental Health Services Trauma, Anxiety, and Depression Clinic, South London and Maudsley National Health Service Foundation Trust, London, UK

**Keywords:** Gait speed, Immunosenescence, Inflammaging, MRI, Pace of aging

## Abstract

**Background:**

To understand and measure the association between chronic inflammation, aging, and age-related diseases, broadly applicable standard biomarkers of systemic chronic inflammation are needed. We tested whether elevated blood levels of the emerging chronic inflammation marker *soluble urokinase plasminogen activator receptor* (suPAR) were associated with accelerated aging, lower functional capacity, and cognitive decline.

**Methods:**

We used data from the Dunedin Study, a population-representative 1972–1973 New Zealand birth cohort (*n* = 1037) that has observed participants to age 45 years. Plasma suPAR levels were analyzed at ages 38 and 45 years. We performed regression analyses adjusted for sex, smoking, C-reactive protein, and current health conditions.

**Results:**

Of 997 still-living participants, 875 (88%) had plasma suPAR measured at age 45. Elevated suPAR was associated with accelerated pace of biological aging across multiple organ systems, older facial appearance, and with structural signs of older brain age. Moreover, participants with higher suPAR levels had greater decline in physical function and cognitive function from childhood to adulthood compared to those with lower suPAR levels. Finally, improvements in health habits between ages 38 and 45 (smoking cessation or increased physical activity) were associated with less steep increases in suPAR levels over those years.

**Conclusions:**

Our findings provide initial support for the utility of suPAR in studying the role of chronic inflammation in accelerated aging and functional decline.

A major public health challenge is to extend healthspan in concert with the lifespan of an ever-expanding aging population (1,2). Systemic chronic inflammation is a major driver of pathogenesis and progression of common, age-related chronic diseases (eg, cardiovascular disease, type 2 diabetes, cancer, and neurodegenerative disorders) (3). To delay the onset of common age-related diseases and extend years lived free of disease and disability, interventions to slow chronic inflammation and accelerated aging must be applied *before* the development of manifest disease. The identification of reliable biomarkers of systemic chronic inflammation is therefore critical.

Although there are several ways to assess inflammation, there are currently no standard biomarkers for indicating the presence of health-damaging chronic inflammation (3). Chronic inflammation is typically measured by combining canonical biomarkers of acute inflammation (3), many of which are short-lived and rapidly up- and down-regulated, which complicates quantification and clinical interpretation. While the acute-phase reactant C-reactive protein (CRP) is commonly used as the gold standard inflammation marker both in the clinic and in life-course research (4), *soluble urokinase plasminogen activator receptor* (suPAR) is a newer biomarker of inflammation (5), which appears to be correlated with chronic rather than acute inflammation. Although CRP and suPAR are positively correlated, they appear to capture different aspects of inflammation (6).

suPAR is the soluble form of the membrane-bound receptor uPAR. It is released to the bloodstream during pro-inflammatory conditions when uPAR is cleaved from the surface of immunologically active cells. The blood concentration of suPAR is thought to reflect a person’s overall level of immune activity, and elevated suPAR is associated with the development, presence, and progression of disease (5,7,8). suPAR levels increase with age (9,10), are elevated across a wide range of diseases (11), including cardiovascular disease (12), type 2 diabetes (13), cancer (14,15), renal disease (16,17), and infections (18), and predict early mortality, both in the general population and in patient populations (7,11). suPAR has also been shown to be associated with psychosocial exposures and health habits. For example, exposure to adverse childhood experiences is associated with elevated suPAR levels later in life, even more so than the pro-inflammatory biomarkers CRP and interleukin-6 (19,20). In addition, poor health habits (eg, unhealthy diet, smoking, and physical inactivity) have been linked to higher suPAR levels (10).

In summary, suPAR may be a biomarker of systemic chronic inflammation since (i) it reflects inflammation and immune activation (its expression and release are upregulated by increased immune activation (21,22), and its blood concentration is positively correlated with established biomarkers of inflammation (19,20,23)); (ii) it shares the same risk factors as many age-related diseases (eg, older age, chronic infections, unhealthy lifestyle, social stressors) (3,10,19,21); and (iii) it predicts (7) and is elevated by age-related diseases (11). But in contrast to many currently used markers of systemic inflammation, suPAR is minimally affected by acute changes and short-term influences (except smoking) (24).

Here, we explored the potential of the emerging chronic inflammation marker suPAR to track accelerated aging. Using data from the Dunedin Study, which has followed a population-representative birth cohort to age 45 years, we tested the hypothesis that elevated suPAR would be associated, already by midlife, with a faster pace of biological aging, lower functional capacity (more physical limitations, poorer physical function), as well as cognitive decline. In addition, in secondary analyses, we tested whether improvements in health habits, observed among some participants from age 38 to 45 years, were associated with slower age-related increases in suPAR levels.

## Method

### Study Design and Population

Participants are members of the Dunedin Study, a longitudinal investigation of health and behavior in a representative birth cohort. Participants (*n* = 1037; 91% of eligible births; 52% male) were all individuals born between April 1972 and March 1973 in Dunedin, New Zealand (NZ), who were eligible based on residence in the province and who participated in the first assessment at age 3 (25). The cohort represented the full range of socioeconomic status (SES) in the general population of NZ’s South Island and as adults matched the NZ National Health and Nutrition Survey on key adult health indicators (eg, body mass index, smoking, GP visits) and the NZ Census of citizens of the same age on educational attainment (26). The cohort is primarily white (93%), matching South Island demographics (25). Assessments were carried out at birth and ages 3, 5, 7, 9, 11, 13, 15, 18, 21, 26, 32, 38, and most recently (completed April 2019) 45 years, when 94.1% (*n* = 938) of the 997 participants still alive took part. At each assessment, each participant was brought to the research unit for interviews and examinations. The relevant ethics committees approved each phase of the study, and informed consent was obtained from all participants.

### Measures of Inflammation

Plasma suPAR (ng/mL) was analyzed at ages 38 and 45 with the suPARnostic AUTO Flex ELISA (ViroGates A/S, Birkerød, Denmark) according to manufacturer’s instructions, as previously described (19). The detection limit of the assay was 0.1 ng/mL. The intraassay correlation of repeat measurements of the same sample was *r* = 0.98 and coefficient of variation (CV) = 2.4%, and the interassay correlation was *r* = 0.81 and CV = 12.8%. Serum high-sensitivity CRP (hsCRP, mg/L) was measured on a Cobas c702 analyzer (Roche Diagnostics GmbH) at age 45, using a particle-enhanced immunoturbidimetric assay.

### Health Habits

*Smoking* was assessed as current smoking, lifetime pack-years, and number of cigarettes per day at ages 38 and 45 years.

*Physical activity* was assessed as sport/leisure-time physical activity at ages 38 and 45 years, as previously described (27). Trained interviewers guided participants through reporting the different types of physically demanding activities they engaged in during an average week and an average weekend. Participants indicated number of minutes spent doing each activity at a moderate or more strenuous level of difficulty. Time spent on each activity was converted to metabolic equivalent (MET) units, with moderate-intensity activity given a weight of 4, hard activity given a weight of 6, and very hard activity given a weight of 10 (28). We summed weekday and weekend METs from moderate or more strenuous leisure activities to calculate physical activity levels at ages 38 and 45. Participants were grouped according to U.S. Department of Health and Human Services Physical Activity Guidelines for Americans (https://health.gov/sites/default/files/2019-09/Physical_Activity_Guidelines_2nd_edition.pdf): at age 45, 31% of the cohort (*n* = 268) was sedentary (ie, they engaged in 0 minutes of moderate or more strenuous leisure-time physical activity per week); 20% (*n* = 173) non-sedentary, but did not achieve the 500 METs/wk minimum recommended dosage of physical activity; 17% (*n* = 146) achieved 500–1000 METs/wk; and 33% (*n* = 287) exceeded 1000 METs/wk.

*Alcohol use* at ages 38 and 45 was assessed as number of drinks per week and categorized according to the national recommendations by the NZ Ministry of Health of maximum 10 drinks per week for women and 15 drinks per week for men (https://www.health.govt.nz/your-health/healthy-living/addictions/alcohol-and-drug-abuse/alcohol).

### Health Measures

Body mass index (kg/m^2^) was measured at age 45 years.

Use of *anti-inflammatory medication* at the time of interview was assessed at age 45 years. Anti-inflammatory medications include non-steroidal anti-inflammatory drugs (NSAIDs), anti-gout medication, corticosteroids (respiratory, systemic), anti-rheumatics, prophylactic aspirin, and statins.

*Self-reported health* was assessed at age 45, by asking respondents: “In general, would you say your health is excellent/very good/good/fair/poor?”.

*Current health conditions* at age 45 were measured as a total count of health conditions based on the Category I domain “Organ System Diseases Diagnosed” from the Comprehensive Model of Health developed by McClintock et al. (29). As detailed in Supplementary Table S1, one point was given for each of 14 different conditions falling within the following 6 domains: (i) endocrine, (ii) cardiovascular, (iii) lung, (iv) immune, (v) filtration, and (vi) cancer. Among participants included at age 45 (*n* = 931), 379 (40.7%) had no conditions, 321 (34.5%) had one, 150 (16.1%) had two, 63 (6.8%) had three, 12 (1.3%) had four, 4 (0.4%) had five, and 2 (0.2%) had 6 conditions. The McClintock Comprehensive Model of Health is consistent with the World Health Organization’s definition of health.

### Measures of Aging, Functional Capacity, and Cognitive Function

*Aging* was assessed by 3 measures: Pace of Aging (30), Facial Age (30), and brain age gap estimate (brainAGE) (31).

*Pace of Aging* was measured for each participant with repeated assessments of a panel of 19 biomarkers taken at ages 26, 32, 38, and 45 years, as previously described (30,32). The 19 biomarkers were: body mass index, waist-hip ratio, HbA1C, leptin, blood pressure (mean arterial pressure), cardiorespiratory fitness (VO_2_Max), FEV_1_, FEV_1_/FVC, total cholesterol, triglycerides, high-density lipoprotein (HDL) cholesterol, apolipoprotein B100/A1 ratio, lipoprotein(a), creatinine clearance, blood urea nitrogen (BUN), CRP, white blood cell count, mean periodontal attachment loss, and caries-affected tooth surfaces. The measurement of each biomarker is described in Supplementary eMethods 1. Change over time in each biomarker was modeled with a mixed-effects growth model, and these 19 rates of change were combined into a single index scaled (within sex) in years of physiological change occurring per 1 chronological year. Participants ranged in their Pace of Aging from 0.4 years of physiological change per chronological year to nearly 2.4 years of physiological change per chronological year.

*Facial Age* at age 45 was based on ratings by an independent panel of 8 raters of each participant’s facial photograph, as previously described (30). Facial Age was based on 2 measurements of perceived age. First, *Age Range* was assessed by an independent panel of 4 raters, who were presented with standardized (non-smiling) facial photographs of participants and were kept blind to their actual age. Raters used a Likert scale to categorize each participant into a 5-year age range (ie, from 20 to 24 years old up to 70+ years old) (interrater reliability = .77). Scores for each participant were averaged across all raters. Second, *Relative Age* was assessed by a different panel of 4 raters, who were told that all photos were of people aged 45 years old. Raters then used a 7-item Likert scale to assign a “relative age” to each participant (1 = “young looking,” 7 = “old looking”) (interrater reliability = .79). The measure of perceived age at 45 years, Facial Age, was derived by standardizing and averaging Age Range and Relative Age scores.

*BrainAGE* at age 45 was derived from structural MRI data collected using a Siemens Skyra 3T scanner (Siemens Healthcare, Erlangen, Germany) equipped with a 64-channel head/neck coil. Specifically, we derived a brainAGE score, as previously described (31), calculated as the difference between a participant’s predicted age from structural MRI data and their exact chronological age, between birth and the date of the MRI scan. We chose the brainAGE algorithm because of its performance in predicting chronological age in independent samples and its sensitivity to age-related cognitive impairment in old age (33). The algorithm is trained on vertex-wise cortical thickness and surface area data as well as subcortical gray matter volume extracted from standard space (see Supplementary eMethods 2 for details). Test–retest reliability of brainAGE was assessed in 20 participants (mean interval between scans = 79 days) and found to be excellent (intraclass correlation coefficient = .81; 95% confidence interval [CI]: 0.59–0.92).

*Functional capacity* at age 45 was assessed by self-reports of physical limitations and by several brief exercises that index the ability to perform everyday activities: one-legged balance, handgrip strength, gait speed, 2-minute step test, and chair-stand test, as previously described (30).

*Physical limitations* were measured with the 10-item RAND 36-Item Health Survey 1.0 physical functioning scale (34). Participant responses (“limited a lot,” “limited a little,” “not limited at all”) assessed their difficulty with completing various activities (eg, climbing several flights of stairs, walking more than 1 km, participating in strenuous sports). Scores were reversed to reflect physical limitations so that a high score indicates more limitations.

*One-legged balance* was measured using the Unipedal Stance Test as the maximum time achieved across 3 trials of the test with eyes closed (35–37).

*Handgrip strength* was measured (elbow held at 90°, upper arm held tight against the trunk) as the maximum value achieved across 3 trials for each hand using a Jamar digital dynamometer (38,39).

*Gait speed* (m/s) was assessed with the 6 m long GAITRite Electronic Walkway (CIR Systems Inc., Franklin, NJ) with 2 m acceleration and 2 m deceleration before and after the walkway, respectively. Gait speed was assessed under 3 walk conditions: usual gait speed (walk at normal pace from a standing start; average of 2 walks) and 2 challenge paradigms, that is, dual-task gait speed (walk at normal pace while reciting alternate letters of the alphabet out loud, starting with the letter “A”; average of 2 walks) and maximum gait speed (walk as fast as safely possible; average of 3 walks). To increase reliable measurement and take advantage of the variation in all 3 walk conditions (usual gait and the 2 challenge paradigms), we averaged the 3 individual walk conditions to generate a primary gait measure of composite gait speed (30).

The *2-minute step test* measured the number of times a participant lifted their right knee to mid-thigh height (measured as the height half-way between the knee cap and the iliac crest) in 2 minutes at a self-directed pace (40,41).

*Chair stands* were measured as the number of stands a participant completed in 30 seconds from a seated position (40,42).

*Cognitive function* was assessed through standardized testing. Childhood cognitive function was assessed by calculating mean scores for the Wechsler Intelligence Scale for Children–Revised (WISC-R) across administration at ages 7, 9, and 11 years. Adulthood cognitive function was assessed with the Wechsler Adult Intelligence Scale–IV (WAIS-IV) (43) administered at age 45 years. Cognitive decline was calculated by a residualized change score between scores on the WISC-R and the WAIS-IV. The WISC-R and the WAIS-IV are ideal for measuring child-to-adult cognitive decline because both tests are matched for content coverage and format, both were individually administered by trained psychometrists, and both yield summary scores that are reliable at >.95.

### Statistical Analysis

Continuous variables are reported as mean (standard deviation [*SD*]) and categorical variables as *n* (%). suPAR was normally distributed, and we used continuous suPAR for analyses. For graphical presentation, we created quintiles with the following cutoffs: Q1, lowest suPAR: ≤2.31 ng/mL (*n* = 175, 20.0%); Q2: 2.31–2.67 ng/mL (*n* = 176, 20.1%); Q3: 2.67–3.04 ng/mL (*n* = 174, 19.9%); Q4: 3.04–3.53 ng/mL (*n* = 175, 20.0%); Q5, highest suPAR: >3.53 ng/mL (*n* = 175, 20.0%). CRP levels were log-transformed for analyses to improve normality of the distribution.

We calculated Pearson’s and Spearman’s correlation coefficients with 95% CIs to test associations between suPAR and measures of lifestyle, health, aging, functional capacity, and cognitive function.

To test associations between suPAR and aging outcomes, we used Ordinary Least Squares regression with suPAR as the dependent variable. As CRP is the current gold standard marker of inflammation, we added CRP to the regression analyses to test if suPAR offered incremental validity. Moreover, as suPAR is associated with chronic disease, we added controls for current health conditions to reflect the underlying health of each participant. Thus, multivariable regression analyses were adjusted for the covariates (i) sex and current smoking, (ii) sex, current smoking, and CRP, and (iii) sex, current smoking, CRP, and current health conditions. We report standardized regression coefficients (βs) with 95% CIs. We further tested the association between high suPAR levels (>3.53 ng/mL; highest suPAR quintile) and aging outcomes using logistic regression, reporting odds ratios with 95%CIs.

In this longitudinal cohort, several measures of physical function were assessed both at ages 38 and 45. To test whether elevated suPAR at age 38 was associated with physical decline, we were able to calculate difference scores (Δ) for the following variables: Facial Age, physical limitations, handgrip strength, and one-legged balance. We regressed the change in outcome on age 38 suPAR controlling for the baseline level of each outcome variable at age 38 years and sex.

In this longitudinal cohort, we observed changes in health habits between age 38 and 45. To test whether improvements in health habits were associated with slower increases in suPAR levels, we calculated change scores (Δ) for measures of smoking (numbers of cigarettes smoked per day), physical activity level (METs per week), and alcohol use (numbers of drinks per week) as well as the change in suPAR level. We regressed ΔsuPAR on change in each health habit, controlling for the baseline level of each health habit at age 38 years and sex.

Statistical analyses were performed in SAS Enterprise Guide (SAS Institute Inc, Cary, NC). Figures were created with GraphPad Prism v.8.0.0 (GraphPad Software, Inc., San Diego, CA) and RStudio v.1.1.456 (RStudio, Boston, MA). Analyses reported here were pre-registered (https://sites.google.com/site/moffittcaspiprojects/) and checked for reproducibility by an independent data analyst, who derived the code by working from the manuscript and applied it to a fresh copy of the data set. A *p* < .05 was designated as statistically significant, and we further report Bonferroni-corrected *p* levels.

## Results

Of 1037 participants in the original cohort, 997 were still alive at age 45 years, and 938 took part in the age-45 assessment between April 2017 and April 2019. Of the 938 who participated, 879 had blood drawn, and 875 (93.3%) had plasma suPAR measured and were included in this study. Participants with suPAR data available were similar to the full cohort at age 45 (Supplementary Table S2).

For 843 participants, suPAR was measured at both ages 38 and 45 years; suPAR levels increased from 2.39 ng/mL (*SD* 0.89) at age 38 to 3.01 (*SD* 1.03) at age 45 years. There was a positive correlation between suPAR measured at age 38 and suPAR measured at age 45 (adjusted for sex): *r* = 0.58 (95% CI 0.53–0.62, *p* < .0001), indicating that individuals tended to retain their rank in the population on suPAR over a period of 7 years.

Mean suPAR levels stratified by cohort characteristics are given in Table 1, along with correlation coefficients between suPAR and these cohort characteristics (equivalent coefficients for CRP are shown in Supplementary Table S3). Women had higher suPAR than men. Tobacco smoking and sedentary lifestyle were associated with elevated suPAR, while alcohol use was not significantly associated with suPAR. Elevated suPAR was associated with higher body mass index and elevated CRP at age 45, but not with the use of anti-inflammatory medication. Participants with poor self-reported health at midlife had higher suPAR, and those suffering from 1 or more current health conditions also had elevated suPAR compared to those without any current health conditions at age 45.

**Table 1.** Mean suPAR Levels (ng/mL) and Correlation Coefficients for Participants in the Dunedin Study at Age 45 Years Stratified by Cohort Characteristics

Variable	*N* (%)	*M* (*SD*)	*r* (95% CI)	*p* Value*
Total *N*	875 (100)	3.03 (1.06)		
Sex	875 (100)		−0.22 (−0.28; −0.15)^†^	<.0001
Female	431 (49.3)	3.18 (0.99)		
Male	444 (50.7)	2.88 (1.10)		
Health habits				
Current smoking	873 (99.8)		0.35 (0.29; 0.40)^†^	<.0001
Nonsmoking	697 (79.8)	2.86 (0.93)		
Smoking	176 (20.2)	3.71 (1.23)		
Physical activity (Mets min/wk)	874 (99.9)		−0.17 (−0.23; −0.10)^‡^	<.0001
500+ Mets min/wk	433 (49.5)	2.88 (0.95)		
<500 Mets min/wk	441 (50.5)	3.18 (1.13)		
Alcohol use (drinks/wk)	872 (99.7)		−0.02 (−0.09; 0.04)^‡^	.47
Within recommendations	608 (69.7)	3.07 (1.12)		
Above recommendations	264 (30.3)	2.94 (0.90)		
Health				
Body mass index (kg/m^2^)	873 (99.8)		0.11 (0.04; 0.18)^‡^	.0011
ln(CRP)^§^	873 (99.8)		0.25 (0.19; 0.31)^‡^	<.0001
Anti-inflammatory medication	875 (100)		0.06 (−0.01; 0.13)^†^	.07
No	625 (71.4)	2.98 (0.90)		
Yes	250 (28.6)	3.15 (1.36)		
Self-reported health	874 (99.9)		−0.27 (−0.33; −0.20)^†^	<.0001
Excellent	151 (17.3)	2.77 (0.74)		
Very good	371 (42.4)	2.83 (0.77)		
Good	274 (31.4)	3.18 (0.94)		
Fair	65 (7.4)	3.72 (1.29)		
Poor	13 (1.5)	5.10 (4.12)		
Current health conditions	874 (99.9)		0.30 (0.24; 0.36)^‡^	<.0001
None	345 (39.5)	2.80 (0.78)		
1+	529 (60.5)	3.18 (1.18)		

*Notes:* CI = confidence interval; CRP = C-reactive protein; *SD* = standard deviation; suPAR = soluble urokinase plasminogen activator receptor.

*Bonferroni-corrected *p* level = .003.

^†^Spearman correlation coefficient.

^‡^Pearson correlation coefficient.

^§^Log-transformed (natural logarithm) serum high-sensitivity CRP.

### Is Elevated suPAR Associated With Accelerated Aging, Lower Functional Capacity, and Poor Cognitive Function in Midlife?

**Figure 1.**
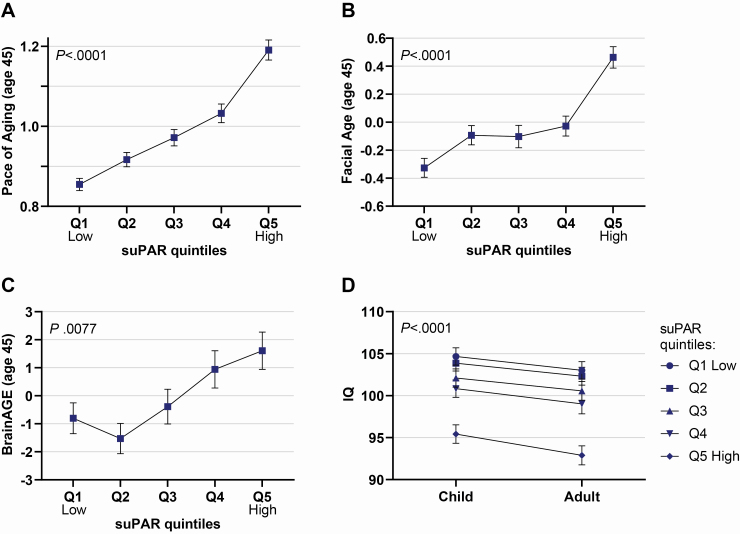
Accelerated aging and cognitive decline are associated with elevated suPAR. (**A**) Mean Pace of Aging (years of physiological change per chronological year), (**B**) mean Facial Age (z-score, mean = 0, *SD* = 1), and (**C**) mean brainAGE by suPAR quintiles at age 45 years (generalized additive models for panel A–C are shown in Supplementary eFigure 1). (**D**) Child-to-adult cognitive decline by suPAR quintiles. Number of participants in each suPAR quintile: Q1: *n* = 175 (≤2.31 ng/mL); Q2: *n* = 176 (2.31–2.67 ng/mL); Q3: *n* = 174 (2.67–3.04 ng/mL); Q4: *n* = 175 (3.04–3.53 ng/mL); Q5: *n* = 175 (>3.53 ng/mL). Error bars indicate standard error. *p* values for comparisons between suPAR quintiles were obtained with the Kruskal–Wallis test. BrainAGE = brain age gap estimate; suPAR = soluble urokinase plasminogen activator receptor.

Participants who exhibited signs of accelerated aging at midlife had elevated suPAR levels (Figure 1; Table 2). Elevated suPAR at age 45 years was associated with a more rapid Pace of Aging from age 26 to 45 years (*r* 0.38 [95% CI 0.32–0.44], *p* < .0001; Figure 1A); participants with the highest suPAR (top quintile) had on average been aging 6.4 years faster than those with the lowest suPAR (bottom quintile) (Figure 1A). In addition, at age 45 years the faces of participants with elevated suPAR were rated as looking older (*r* 0.27 [95% CI 0.21–0.33], *p* < .0001; Figure 1B), and their brains exhibited structural signs of older brainAGE (*r* 0.15 [95% CI 0.08–0.21], *p* < .0001; Figure 1C).

**Table 2.** Associations of Age 45 Measures of Aging, Functional Capacity, and Cognitive Function With Plasma suPAR Levels at Age 45 in *n* = 875 Participants in the Dunedin Study*

Variable	Adjusted for Sex and Smoking			Adjusted for Sex, Smoking, and ln(CRP)			Adjusted for Sex, Smoking, ln(CRP), and Current Health Conditions		
	*N*	β (95% CI)*	*p* Value^†^	*N*	β (95% CI)*	*p* Value^†^	*N*	β (95% CI)*	*p* Value^†^
Aging									
Pace of Aging	872	.32 (.26; .38)	<.0001	870	.28 (.21; .35)	<.0001	870	.22 (.15; .29)	<.0001
Facial Age	871	.19 (.13; .26)	<.0001	869	.16 (.10; .22)	<.0001	869	.13 (.07; .19)	<.0001
BrainAGE	841	.08 (.02; .15)	.012	839	.06 (−.0002; .13)	.051	839	.06 (−.002; .12)	.056
Functional capacity									
Physical limitations	870	.27 (.21; .33)	<.0001	868	.24 (.18; .30)	<.0001	868	.20 (.13; .26)	<.0001
One-legged balance	865	−.15 (−.21; −.09)	<.0001	863	−.11 (−.17; −.04)	.0010	863	−.07 (−.13; −.01)	.024
Handgrip strength	872	−.18 (−.29; −.08)	.0004	870	−.16 (−.26; −.06)	.0017	870	−.14 (−.24; −.04)	.0051
Composite gait speed^‡^	871	−.18 (−.24; −.11)	<.0001	869	−.14 (−.20; −.08)	<.0001	869	−.11 (−.17; −.05)	.0005
2-min step test	854	−.11 (−.16; −.04)	.0006	852	−.08 (−.13; −.01)	.019	852	−.05 (−.10; .01)	.11
Chair stands	843	−.17 (−.21; −.10)	<.0001	841	−.13 (−.17; −.06)	<.0001	841	−.10 (−.15; −.04)	.0011
Cognitive function									
Adult IQ	871	−.17 (−.23; −.10)	<.0001	869	−.14 (−.20; −.08)	<.0001	869	−.11 (−.18; −.05)	.0003
Child-to-adult cognitive decline	860	−.08 (−.14; −.02)	.012	858	−.07 (−.13; −.01)	.022	858	−.06 (−.12; .004)	.066

*Notes:* BrainAGE = brain age gap estimate; CI = confidence interval; CRP = C-reactive protein; suPAR = soluble urokinase plasminogen activator receptor.

*Standardized β coefficients.

^†^Bonferroni-corrected *p* level = .004.

^‡^Gait speed was measured as an average across the 3 individual walk conditions (usual, dual task, and maximum gait speed) to generate the measure of composite gait speed.

suPAR was also associated with measures of functional capacity at age 45 (Table 2). Participants who self-reported more physical limitations had higher suPAR (*r* 0.32 [95% CI 0.26–0.38], *p* < .0001). In addition, participants with poorer balance (*r* −0.20, 95% CI −0.27 to −0.14), weaker grip strength (*r* −0.19, 95% CI −0.25 to −0.12), slower gait speed (*r* −0.23, 95% CI −0.30 to −0.17), and those who performed worse on the 2-minute step test (*r* −0.18, 95% CI −0.24 to −0.11) and the chair-stand test (*r* −0.23, 95% CI −0.29 to −0.16) had higher suPAR levels (all *p* < .0001).

Next, we tested if neurocognitive functioning at age 45 was also associated with suPAR. Participants with lower IQ at age 45 had higher suPAR (*r* −0.25 [95% CI −0.31 to −0.18], *p* < .0001; Figure 1D). Participants with higher suPAR at age 45 also exhibited a bigger decline in cognitive functioning from childhood to adulthood than those with lower suPAR levels (Figure 1D; Table 2).

The associations between elevated suPAR with all measures of accelerated aging, lower functional capacity, poorer cognitive functioning, and cognitive decline held after controlling for sex, smoking, CRP, and current health conditions (Table 2) with the exception of the 2-minute step test and trending associations for brainAGE (*p* = .056) and cognitive decline (*p* = .066). Supplementary Table S4 compares associations for CRP and suPAR with these measures. Association analyses were also repeated, comparing participants with elevated suPAR (highest suPAR quintile) to the remaining participants (Supplementary Table S5).

### Is Higher suPAR Associated With Physical Decline?

**Figure 2.**
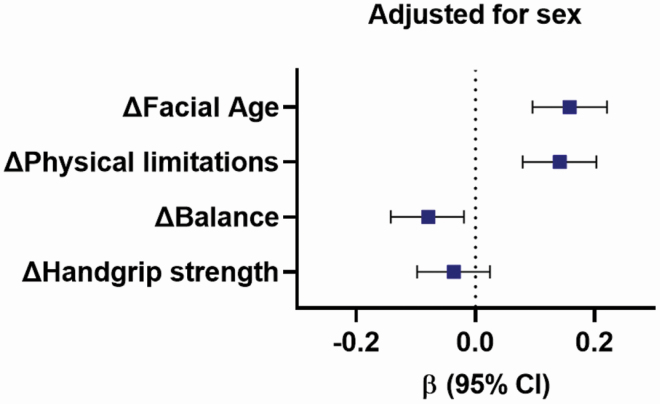
suPAR at age 38 is associated with physical decline from age 38 to 45. Associations (standardized β coefficients with 95% confidence intervals) of suPAR measured at age 38 with change in Facial Age, physical limitations (RAND SF36 physical functioning scale), one-legged balance, and handgrip strength from age 38 to 45. Associations were adjusted for the age 38 level of each outcome measure and sex. Change was measured as a difference score between age 45 and age 38. suPAR = soluble urokinase plasminogen activator receptor.

Next, we tested if suPAR measured 7 years earlier, at age 38 years, was associated with a physical decline during the intervening period from age 38 to 45 years. Participants with elevated suPAR at age 38 exhibited accelerated facial aging (β .16, 95% CI .10–.22, *p* < .0001), more physical limitations (β .14, 95% CI .08–.20, *p* < .0001), and decline in one-legged balance (β −.08, 95% CI −.14 to −.02, *p* = .010), but no change in handgrip strength (Figure 2).

### Do Improvements in Health Habits Associate With a Decrease in suPAR?

**Figure 3.**
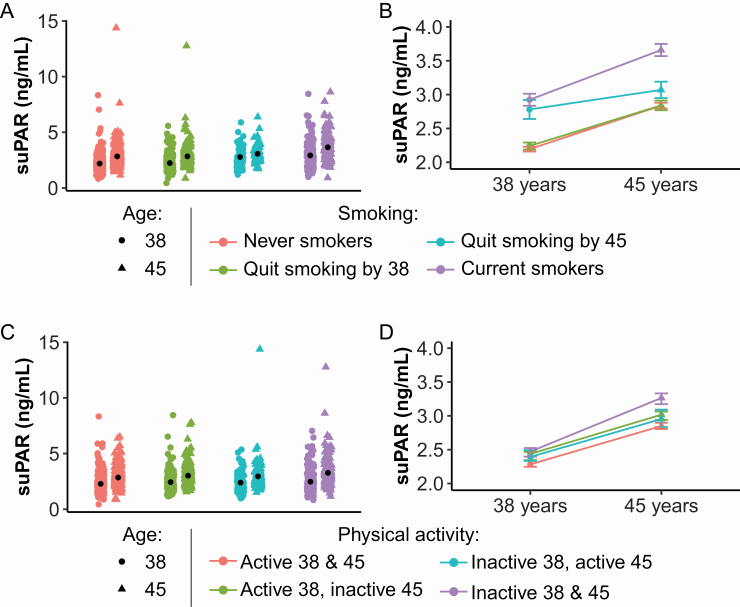
Change in health habits is associated with change in suPAR levels. suPAR levels by smoking (**A** and **B**, *n* = 842) and sports/leisure-time physical activity (**C** and **D**, *n* = 840) categories at ages 38 and 45 years. For smoking (**A** and **B**), participants were categorized as those who never smoked (*n* = 424); those who were former smokers but quit before age 38 (*n* = 203); those who were former smokers but quit between age 38 and age 45 (*n* = 52); and those who were still smoking at age 45 (*n* = 163). For physical activity (**C** and **D**), participants were categorized as those who were physically active (ie, achieved the minimum recommended dosage of physical activity of 500 METs per week) at both ages 38 and 45 (*n* = 311); those who were physically active at age 38 but inactive (ie, did not achieve 500 METs per week) at age 45 (*n* = 221); those who were inactive at age 38 but physically active at age 45 (*n* = 197); and those who were physically inactive at both ages 38 and 45 (*n* = 111). Panels **A** and **C** show scatterplots of suPAR levels at age 38 and 45 years with black dots indicating mean suPAR. Panels **B** and **D** show mean suPAR at age 38 and 45 years with error bars indicating standard error. Note: The 2 individuals with suPAR > 10 ng/mL both had kidney disease and were undergoing dialysis. Controls for kidney disease among other conditions did not alter the findings, see Table 2. suPAR = soluble urokinase plasminogen activator receptor.

Finally, we investigated whether participants who had improved their health habits exhibited a smaller age-related increase in suPAR. For participants who smoked (Figure 3A and B), a decrease in the number of cigarettes smoked per day from age 38 to 45 was associated with slower increases in suPAR level (β .12, 95% CI .04–.20, *p* = .0049). Similarly, for physical activity (Figure 3C and D), an increase in METs per week from age 38 to 45 was associated with slower increases in suPAR level (β −.14, 95% CI −.23 to −.04, *p* = .0058). For alcohol use (Supplementary eFigure 2), there was no significant association between a decrease in alcohol use from age 38 to 45 and change in suPAR level (β .06, 95% CI −.02 to .12, *p* = .13).

## Discussion

suPAR is an emerging biomarker of systemic chronic inflammation. In this longitudinal study of a population-representative birth cohort, we tested the association of suPAR with midlife indicators of accelerated aging, functional and cognitive decline, as well as with lifestyle changes from age 38 to 45.

First, elevated suPAR was associated with accelerated aging at midlife, as indexed by more rapid decline of multiple organ systems over the preceding 2 decades (Pace of Aging), by older-looking facial age, by signs of older structural brainAGE, and by decline in neurocognitive functioning from childhood to midlife. Elevated suPAR was also associated with multiple indicators of worsened functional capacity, including more physical limitations, poorer balance, weaker grip strength, slower gait speed, and poorer performance on the 2-minute step test and the chair-stand test. These associations between elevated suPAR and accelerated aging were not simply an artifact of current poor health, as evidenced in analyses controlling for current health conditions. Moreover, suPAR appears to add information about aging beyond the established biomarker CRP; suPAR remained associated with aging outcomes in analyses controlling for CRP, and in addition, effect sizes were bigger for suPAR than for CRP.

Second, elevated suPAR measured at age 38 was associated with physical decline (accelerated facial aging, more physical limitations, and decline in one-legged balance) during the intervening period from age 38 to 45 years, underscoring the potential prognostic value of this biomarker of inflammation. The accelerated aging process and physical decline found in middle-aged individuals with high suPAR levels might lead to increased frailty later in life.

Third, elevated suPAR was associated with an unhealthy lifestyle at midlife. But improvements in health habits from age 38 to 45 years were mirrored in smaller increases in suPAR levels, such that those who quit or reduced their tobacco smoking or increased their physical activity level did not increase as much in suPAR with age as those who did not improve their health habits. Interestingly, participants who stopped smoking before age 38 appeared to have suPAR levels similar to those who had never smoked. This is in line with previous observational and smoking-cessation studies (10,44). Low levels of physical activity have also previously been shown to be associated with higher levels of suPAR (10). Similar to previous findings, we found no association between change in alcohol use and change in suPAR (10).

Systemic chronic inflammation can arise with advanced aging, due to the progressive age-related changes of the immune system, so-called *immunosenescence* and *inflammaging*. Immunosenescence is the gradual decline of the immune system, which is accelerated by prolonged antigenic stimulation over the course of life, resulting in increased susceptibility to infections, neoplasias, and autoimmune manifestations. Moreover, immunosenescence leads to accelerated inflammaging, that is, elevated secretion of pro-inflammatory cytokines and reduction of anti-inflammatory cytokines (45). These dysregulated immunological phenotypes—immunosenescence and inflammaging—are recognized as hallmarks of aging (46). As they directly affect tissue homeostasis and result in age-related functional decline, they have detrimental effects, such as causing systemic chronic inflammation and affecting metabolism, vascular aging, neurological and cognitive functions, and muscle- and bone metabolism. Thus, immunosenescence, inflammaging, and chronic systemic inflammation can all contribute to accelerated frailty and progression of age-related chronic diseases (45,47,48). Despite the vast number of immunological mediators involved in immunosenescence and inflammaging, it has remained a challenge to identify biomarkers of the aged immune system that are broadly applicable and show stable clinical associations across different populations (49).

Our findings of associations between suPAR and multiple indicators of aging and functional decline provide further support for the theory of immunosenescence and inflammation in aging. While many inflammatory biomarkers have been shown to increase with age (CRP, interleukin-6, tumor necrosis factor [TNF]-α) (45,50), conflicting evidence exists and these biomarkers are not consistently found to be elevated in older adults (3,51). Moreover, contrasting findings even suggest that the process of immune aging is context-dependent, with different clinical associations reported in different study populations (49,52). Thus, chronic inflammation and inflammaging are still inadequately estimated by combining markers of acute inflammation, underlining the need to identify actual systemic chronic inflammation biomarkers, which will, in turn, provide useful and predictive information for quantifying age-related disease risk (3).

The findings of this study support the role of suPAR as a biomarker of systemic chronic inflammation and inflammaging; in the present study of midlife adults, suPAR tracked aging well before age-related disease manifested. Along with previously established characteristics, this points to suPAR as a biomarker of chronic rather than acute inflammation, which may provide a new method for assessing systemic chronic inflammation, or even immunosenescence. Randomized clinical trials of anti-aging interventions intended to slow the course of aging could include chronic inflammation biomarkers, such as suPAR, as outcome measures. Assessing suPAR in midlife may also create an opportunity for prevention, as high-risk individuals with elevated chronic inflammation could be identified.

Various multidimensional and multi-omics approaches have recently been investigated as potential measures of systemic chronic inflammation. These include deep molecular profiling of whole-blood transcriptomes, immune proteins, and cell subset frequencies (3,53). However, in contrast to these complex measures, a major advantage of suPAR is that it can easily be measured in plasma or serum at low cost.

### Limitations

The results reported here were based on data from a well-characterized, population-representative birth cohort, with suPAR measured at 2 time points. However, the study has limitations. First, the cohort is predominantly NZ European. Replications are needed in diverse populations. Second, since blood was not biobanked during childhood, we were unable to investigate changes in suPAR levels from childhood to midlife. Longitudinal studies of suPAR over the life-course are needed. Third, we lack detailed information on dietary habits, which have been shown to be associated with suPAR (54). Fourth, the detected effect sizes for suPAR were modest, although this is to be expected in a general population of generally healthy persons at midlife. Fifth, although the distributional properties of suPAR are appealing for research purposes, the optimal threshold for its use as a clinical biomarker has not yet been determined. Sixth, we were able to identify factors associated with elevated suPAR levels, but this observational study design cannot rule out non-causal alternative explanations. Seventh, our findings raise the question of how suPAR specifically relates to hallmarks of aging (46): genomic instability, telomere attrition, epigenetic alterations, loss of proteostasis, deregulated nutrient sensing, mitochondrial dysfunction, cellular senescence, stem cell exhaustion, and altered intercellular communication. Determining the mechanism through which suPAR relates to aging will be an important next step in evaluating the usefulness of suPAR in the study of aging.

## Conclusion

A recent review identified the need for new measures of systemic chronic inflammation to be used to quantify age-related disease risk and to study aging (3). Here we provide initial evidence for the utility of suPAR—an emerging biomarker of systemic chronic inflammation—as an indicator of accelerated aging and functional decline in midlife. We hope that this biomarker will invigorate research in immunoaging well before the onset of age-related diseases and when interventions may have maximal effects.

## Supplementary Material

glaa178_suppl_Supplementary_Material
