# Supplementary material for: Association Between Elevated suPAR, a New Biomarker of Inflammation, and Accelerated Aging
Source: J Gerontol A Biol Sci Med Sci. 2020 Jul 16;76(2):318–27. doi: 10.1093/gerona/glaa178 (PMC7812430; doi:10.1093/gerona/glaa178)
Supplement: glaa178_suppl_Supplementary_Material [file glaa178_suppl_supplementary_material.docx]

**SUPPLEMENT**

**ASSOCIATION BETWEEN ELEVATED suPAR, A NEW BIOMARKER OF CHRONIC INFLAMMATION, AND ACCELERATED AGING**

**Rasmussen et al.**

eMethods 1. Pace of Aging (age 45).

eMethods 2. MRI acquisition and brainAGE.

eTable 1. Domains of health measures and self-reported diagnoses in the current health conditions measure at age 45.

eTable 2. Cohort characteristics at age 45 years for all participants included at age 45, participants with suPAR measured at age 45, or participants with suPAR measured at both age 38 and age 45.

eTable 3. Correlation coefficients for log-transformed C-reactive protein, ln(CRP), with cohort characteristics for Study members in the Dunedin Study at age 45 years.

eTable 4. Associations of age 45 measures of aging, functional capacity, and cognitive function with serum CRP (log-transformed) or plasma suPAR levels at age 45 in n=875 Study members in the Dunedin Study.

eTable 5. Associations between age-45 measures of aging, functional capacity, and cognitive function and high suPAR levels.

eFigure 1. Accelerated Aging is Associated with Elevated suPAR at Age 45 Years.

eFigure 2. suPAR Levels by Alcohol Use (n=838).

References

### **eMethods 1. Pace of Aging (age 45)**

Pace of Aging was measured for each Dunedin participant with repeated assessments of a panel of 19 biomarkers taken at ages 26, 32, 38, and 45 years; a method previously described (1). The 19 biomarkers were: body mass index, waist-hip ratio, glycated hemoglobin (HbA1C), leptin, blood pressure (mean arterial pressure), cardiorespiratory fitness (VO_2_Max), forced expiratory volume in one second (FEV_1_), FEV_1_ to forced vital capacity ratio (FEV_1_/FVC), total cholesterol, triglycerides, high-density lipoprotein (HDL) cholesterol, apolipoprotein B100/A1 ratio, lipoprotein(a), creatinine clearance, blood urea nitrogen (BUN), C-reactive protein, white blood cell count, mean periodontal attachment loss, and caries-affected tooth surfaces. Measures were taken in counterbalanced order across participants with the exception of blood, which was drawn at the same time of day for all participants at all four ages and dental examinations which were conducted in the late afternoon at all four ages. Women who were pregnant at the time of a given assessment were excluded from that wave of data collection. The measurement of each biomarker is described below. Change over time in each biomarker was modeled with mixed-effects growth models, and these rates of change were combined into a single index scaled (within sex) in years of physiological change occurring per one chronological year, as per the method previously described (1). Participants ranged in their Pace of Aging from near 0 years of physiological change per chronological year to nearly 3 years of physiological change per chronological year.

| ***Body mass index*** | Height was measured to the nearest millimeter using a portable stadiometer (Harpenden; Holtain, Ltd.). Weight was measured to the nearest 0.1 kg using calibrated scales. Individuals were weighed in light clothing. Body mass index (BMI) was calculated. |
| --- | --- |
| ***Waist-hip ratio*** | Waist girth was the perimeter at the level of the noticeable waist narrowing located between the costal border and the iliac crest. Hip girth was taken as the perimeter at the level of the greatest protuberance and at about the symphysion pubic level anteriorly. Measurements were repeated and the average used to calculate waist-hip ratio. |
| ***Glycated hemoglobin (HbA1C)*** | Whole blood glycated hemoglobin concentration (expressed as a percentage of total hemoglobin) was measured by ion exchange high performance liquid chromatography (Variant II: BioRad, Hercultes, Calif.), a method certified by the US National Glycohemoglobin Standardization Program (<http://www.ngsp.org/>). |
| ***Leptin*** | Serum leptin (μg/L) was measured using the Quantikine ELISA Human Leptin Immunoassay (Cat# SLP00, R&D Systems Inc, Minneapolis, MN) according to the manufacturer’s instruction. |
| ***Blood pressure (mean arterial pressure)*** | Systolic and diastolic blood pressure were assessed according to standard protocols with a Hawksley random-zero sphygmomanometer with a constant deflation valve. Mean arterial pressure (MAP) was calculated using the formula Diastolic Pressure+1/3(Systolic Pressure - Diastolic Pressure). |
| ***Cardiorespiratory fitness (VO_2_Max)*** | Cardiorespiratory fitness was assessed by measuring heart rate in response to a submaximal exercise test on a friction-braked cycle ergometer. Dependent on the extent to which heart rate increased during a 2-min 50 W warm-up, the workload was adjusted to elicit a steady heart-rate in the range 130–170 beats per minute. After a further 6-min constant power output stage, the maximum heart rate was recorded and used to calculate predicted maximum oxygen uptake adjusted for body weight in milliliters per minute per kilogram (VO_2_max) according to standard protocols (2). |
| ***Lung function (FEV_1_ and*** ***FEV_1_/FVC)*** | We calculated post-albuterol forced expiratory volume in one second (FEV_1_) and the ratio of FEV_1_ to forced vital capacity (FVC; FEV_1_/FVC) using measurements from spirometry conducted with a Sensormedics body plethysmograph (Sensormedics Corporation, Yorba Linda, CA, USA). |
| ***Total cholesterol, triglycerides, and high-density lipoprotein (HDL) cholesterol*** | Serum non-fasting total cholesterol, triglycerides, and high-density lipoprotein (HDL) cholesterol levels (mmol/L) were measured by colorimetric assay on a Hitachi 917 analyzer (ages 26-32), a Modular P analyzer (age 38), and a Cobas c702 analyzer (age 45). |
| ***Apolipoprotein B100/A1 ratio*** | Serum apolipoprotein A1 and apolipoprotein B100 (g/L) were measured by immunoturbidimetric assay on a Hitachi 917 analyzer (ages 26-32), a Modular P analyzer (age 38), and a Cobas c502 (age 45), and the ratio between the two was calculated. |
| ***Lipoprotein(a)*** | Serum lipoprotein(a) (nmol/L) was measured by a particle-enhanced immunoturbidimetric assay on a Hitachi 917 analyzer (ages 26-32), a Modular P analyzer (age 38), and a Cobas c502 analyzer (age 45). |
| ***Creatinine clearance*** | Serum creatinine (mmol/L) was measured by kinetic colorimetric assay on a Hitachi 917 analyzer (age 32), Modular P analyzer (age 38), and Cobas c702 (age 45) (Roche Diagnostics, Mannheim, Germany). For Pace of Aging analysis, creatinine was measured as creatinine clearance, calculated using the National Kidney Foundation CKD-EPI Creatinine Equation (2009) (3). |
| ***Blood urea nitrogen*** | Serum urea nitrogen (mmol/L) was measured by kinetic UV assay at ages 26 (Hitachi 917 analyzer) and 45 (Cobas c702 analyzer), and by kinetic colorimetric assay at ages 32 (Hitachi 917 analyzer) and 38 (Modular P analyzer). |
| ***High sensitivity C-reactive protein (hsCRP)*** | Serum C-reactive protein (mg/L) was measured by high sensitivity immunoturbidimetric assay on a Hitachi 917 analyzer (age 32), a Modular P analyzer (age 38), and a Cobas c702 (age 45). Values were log-transformed for analysis. |
| ***White blood cell count*** | Whole blood white blood cell counts (x10^9^/L) were measured by flow cytometry with a Coulter STKS (Coulter Corporation, Miami, FL) (age 26), a Sysmex XE2100 (Sysmex Corporation, Japan) (age 32), and a Sysmex XE5000 (Sysmex Corporation, Japan) (ages 38 and 45). Counts were log-transformed for analysis. |
| ***Mean periodontal attachment loss*** | Calibrated dentists examined periodontal health at three sites (mesiobuccal, buccal, and distolingual) per tooth. Gingival recession (the distance in millimeters from the cementoenamel junction to the gingival margin) and probing depth (the distance from the probe tip to the gingival margin) were recorded using a PCP-2 periodontal probe (Hu-Friedy; Chicago). The combined attachment loss for each site was computed by summing gingival recession and probing depth (third molars were not included) and then averaged across all periodontally examined teeth. |
| ***Caries-affected tooth surfaces*** | Teeth were examined for caries and restorations following the World Health Organization Oral Health Surveys methodology (4,5). Four surfaces were considered for anterior teeth (canines and incisors): buccal, lingual, distal, and mesial; a fifth surface, occlusal, was considered for premolar and molar teeth. Tooth surfaces were classified as having untreated caries (DS) if a cavitated carious lesion was present, as filled (FS) if a dental restoration was present (including crowns), and missing due to caries (MS) if the participant indicated that a given tooth had been removed due to decay or failed dental restorative work. DS, MS, and FS counts were summed (ranging from 0 to 148 surfaces). Surfaces of teeth that were unerupted, lost due to trauma, extracted for reasons other than caries (impaction, orthodontic treatment, or periodontal disease), or could not be visualized by the examiner were excluded from calculations. |

**eMethods 2. MRI acquisition and brainAGE**

*Image Acquisition*. Each participant was scanned using a Siemens Skyra 3T scanner equipped with a 64-channel head/neck coil at the Pacific Radiology imaging center in Dunedin, New Zealand. High resolution T1-weighted images were obtained using an MP-RAGE sequence with the following parameters: TR = 2400 ms; TE = 1.98 ms; 208 sagittal slices; flip angle, 9°; FOV, 224 mm; matrix =256×256; slice thickness = 0.9 mm with no gap (voxel size 0.9×0.875×0.875 mm); and total scan time = 6 min and 52 s. 3D fluid-attenuated inversion recovery (FLAIR) images were obtained with the following parameters: TR = 8000 ms; TE = 399 ms; 160 sagittal slices; FOV = 240 mm; matrix = 232×256; slice thickness = 1.2 mm (voxel size 0.9×0.9×1.2 mm); and total scan time = 5 min and 38 s. Additionally, a gradient echo field map was acquired with the following parameters: TR = 712 ms; TE = 4.92 and 7.38 ms; 72 axial slices; FOV = 200 mm; matrix = 100×100; slice thickness = 2.0 mm (voxel size 2 mm isotropic); and total scan time = 2 min and 25 s.

*Image Processing*. Structural MRI data were analyzed using the Human Connectome Project (HCP) minimal preprocessing pipeline as extensively detailed elsewhere (6). Briefly, T1-weighted and FLAIR images were processed through the PreFreeSurfer, FreeSurfer, and PostFreeSurfer pipelines. T1-weighted and FLAIR images were corrected for readout distortion using the gradient echo field map, coregistered, brain-extracted, and aligned together in the native T1 space using boundary-based registration (7). Images were then processed with a custom FreeSurfer recon-all pipeline that is optimized for structural MRI with higher resolution than 1 mm isotropic. Finally, recon-all output were converted into CIFTI format and registered to common 32k_FS_LR mesh using MSM-sulc (8). Outputs of the minimal preprocessing pipeline were visually checked for accurate surface generation by examining each participant’s myelin map, pial surface, and white matter boundaries.

For brain volume, cortical thickness, and surface area data, participants were excluded if they failed visual inspection of surface generation, had major incidental findings, or were missing T2-weighted or field map scans, yielding 861 datasets for analyses.

*BrainAGE.* We generated brainAGE scores using a recently published, publicly-available algorithm (9), as previously described (10). This method uses a stacked algorithm to predict chronological age from multiple measures of brain structure derived from Freesurfer version 5.3. Specifically, the algorithm is trained on vertex-wise cortical thickness and surface area data extracted from fsaverage4 standard space as well as subcortical volume extracted from the aseg parcellation. Test-retest reliability was assessed in 20 participants (mean interval between scans = 79 days). The ICC of brainAGE was .81 (95% CI: 0.59–0.92), indicating excellent reliability. We report results of brainAGE calculated as the difference between an individual’s predicted age from MRI data and their exact chronological age, between birth and the date of the MRI scan.

| **eTable 1. Domains of health measures and self-reported diagnoses in the current health conditions measure at age 45** | |
| --- | --- |
| **Domains** | **Comprehensive health measures** |
| **Endocrine** | 1. Diabetes or glycated hemoglobin (HbA1C) >6.5% |
|  | 1. Thyroid |
| **Cardiovascular** | 1. Hypertension, self-reported or systolic blood pressure ≥140 mmHg or diastolic blood pressure ≥90 mmHg |
|  | 1. Rapid pulse ≥80 bpm |
|  | 1. Heart attack, ever |
|  | 1. Cerebrovascular disease, ever |
|  | 1. Heart failure, ever |
| **Lung** | 1. Chronic obstructive pulmonary disease, self-reported or forced expiratory volume in one second (FEV1) to forced vital capacity (FVC) ratio (FEV1/FVC) <70% |
|  | 1. Current asthma |
| **Immune** | 1. Arthritis |
|  | 1. Peptic ulcer |
| **Filtration** | 1. Chronic kidney disease |
|  | 1. Severe liver damage |
| **Cancer** | 1. Any cancer since age 38 |

Notes: One point was given for each of the 14 health measures or self-reported diagnoses within the six domains to generate a score for current health conditions.

| **eTable 2. Cohort characteristics at age 45 years for all participants included at age 45, participants with suPAR measured at age 45, or participants with suPAR measured at both age 38 and age 45** | | | | | | | | | | | | | | | |  |
| --- | --- | --- | --- | --- | --- | --- | --- | --- | --- | --- | --- | --- | --- | --- | --- | --- |
|  | **All seen at age 45** | |  | **suPAR at age 45** | |  | |  | | **suPAR at age 38 & 45** | | | | | |  |
| **Variable** | **N (%)** | **Mean (SD)** |  | **N (%)** | **Mean (SD)** | ***P*** | |  | | **N (%)** | | **Mean (SD)** | | ***P*** | |  |
| Total N | 938 (100) |  |  | 875 (93.3) |  |  | |  | | 843 (89.9) | |  | |  | |  |
| Sex |  |  |  |  |  |  | |  | |  | |  | |  | |  |
| Female | 464 (49.5) |  |  | 431 (49.3) |  |  | |  | | 416 (49.3) | |  | |  | |  |
| Male | 474 (50.5) |  |  | 444 (50.7) |  | .90 | |  | | 427 (50.7) | |  | | .94 | |  |
| **Lifestyle** |  |  |  |  |  |  | |  | |  | |  | |  | |  |
| Current smoking | 923 (98.4) |  |  | 873 (99.8) |  |  | |  | | 841 (99.8) | |  | |  | |  |
| Non-smokers | 724 (78.4) |  |  | 697 (79.8) |  |  | |  | | 678 (80.6) | |  | |  | |  |
| Smokers | 199 (21.6) |  |  | 176 (20.2) |  | .30 | |  | | 163 (19.4) | |  | | .11 | |  |
| Physical activity | 908 (96.8) | 849 (1086) |  | 874 (99.9) | 854 (1086) | .89 | |  | | 842 (99.9) | | 874 (1097) | | .51 | |  |
| 500+ Mets min/week | 446 (49.1) | 1608 (1113) |  | 433 (49.5) | 1606 (1110) |  | |  | | 424 (50.4) | | 1618 (1118) | |  | |  |
| <500 Mets min/week | 462 (50.9) | 116 (166) |  | 441 (50.5) | 116 (165) |  | |  | | 418 (49.6) | | 119 (166) | |  | |  |
| Alcohol use (drinks/week) | 924 (98.5) | 13.2 (20.3) |  | 872 (99.7) | 12.9 (18.9) | .58 | |  | | 840 (99.6) | | 12.9 (19.0) | | .66 | |  |
| Within recommendations | 639 (69.2) | 5.1 (3.7) |  | 608 (69.7) | 5.1 (3.7) |  | |  | | 584 (69.5) | | 5.2 (3.7) | |  | |  |
| Above recommendations | 285 (30.8) | 31.5 (28.7) |  | 264 (30.3) | 30.6 (26.3) |  | |  | | 256 (30.5) | | 30.6 (26.5) | |  | |  |
| **Health** |  |  |  |  |  |  | |  | |  | |  | |  | |  |
| Body mass index (kg/m^2^) | 920 (98.1) | 28.4 (5.8) |  | 873 (99.8) | 28.5 (5.8) | .57 | |  | | 841 (99.8) | | 28.5 (5.8) | | .71 | |  |
| C-reactive protein (mg/L) | 879 (93.7) | 2.72 (5.62) |  | 873 (99.8) | 2.67 (5.47) | .77 | |  | | 841 (99.8) | | 2.67 (5.54) | | .79 | |  |
| Anti-inflammatory medication | 938 (100) |  |  | 875 (100) |  |  | |  | | 843 (100) | |  | |  | |  |
| No | 670 (71.4) |  |  | 625 (71.4) |  |  | |  | | 600 (71.2) | |  | |  | |  |
| Yes | 268 (28.6) |  |  | 250 (28.6) |  | 1.00 | |  | | 243 (28.8) | |  | | .87 | |  |
| Self-reported health | 931 (99.3) | 3.66 (0.90) |  | 874 (99.9) | 3.67 (0.90) | .81 | |  | | 842 (99.9) | | 3.68 (0.89) | | .45 | |  |
| Excellent | 157 (16.9) |  |  | 151 (17.3) |  |  | |  | | 146 (17.3) | |  | |  | |  |
| Very good | 397 (42.6) |  |  | 371 (42.5) |  |  | |  | | 367 (43.6) | |  | |  | |  |
| Good | 293 (31.5) |  |  | 274 (31.4) |  |  | |  | | 256 (30.4) | |  | |  | |  |
| Fair | 70 (7.5) |  |  | 65 (7.4) |  |  | |  | | 61 (7.2) | |  | |  | |  |
| Poor | 14 (1.5) |  |  | 13 (1.5) |  |  | |  | | 12 (1.4) | |  | |  | |  |
| Current health conditions | 931 (99.3) | 0.96 (1.04) |  | 874 (99.9) | 0.97 (1.04) | .61 | |  | | 842 (99.9) | | 0.98 (1.04) | | .57 | |  |
| None | 379 (40.7) | 0 (0) |  | 345 (39.5) | 0 (0) |  | |  | | 330 (39.2) | | 0 (0) | |  | |  |
| 1+ | 552 (59.3) | 1.6 (0.9) |  | 529 (60.5) | 1.6 (0.9) |  | |  | | 512 (60.8) | | 1.6 (0.9) | |  | |  |
| **Aging** |  |  |  |  |  |  | |  | |  | |  | |  | |  |
| Pace of Aging | 932 (99.4) | 1.00 (0.30) |  | 874 (99.9) | 0.99 (0.30) | .63 | |  | | 843 (100) | | 0.99 (0.29) | | .33 | |  |
| Facial Age | 906 (96.6) | 0 (1) |  | 872 (99.7) | -0.02 (0.99) | .61 | |  | | 840 (99.6) | | -0.04 (0.98) | | .21 | |  |
| BrainAGE | 869 (92.6) | 0.00 (8.01) |  | 841 (96.1) | -0.05 (7.97) | .86 | |  | | 811 (96.2) | | -0.11 (7.90) | | .70 | |  |
| **Table continues on next page** | | | | | | | | | | | | | | | |  |
| **eTable 2 continued** | | | | | | | | | | | | | | | | |
| **Functional capacity** |  |  |  |  |  |  |  | |  | |  | |  | |  | |
| Physical limitations | 923 (98.4) | 10.5 (16.4) |  | 871 (99.5) | 10.3 (16.0) | .71 | |  | | 839 (99.5) | | 10.0 (15.6) | | .37 | |  |
| None | 400 (43.3) | 0 (0) |  | 382 (43.9) | 0 (0) |  | |  | | 369 (44.0) | | 0 (0) | |  | |  |
| 1+ | 523 (56.7) | 18.5 (18.1) |  | 489 (56.1) | 18.4 (17.6) |  | |  | | 470 (56.0) | | 17.9 (17.1) | |  | |  |
| One-legged balance, s | 911 (97.1) | 14.7 (9.8) |  | 866 (99.0) | 14.9 (9.9) | .51 | |  | | 835 (99.1) | | 15.1 (9.9) | | .30 | |  |
| Hand grip strength, kg | 920 (98.1) | 39.9 (12.1) |  | 873 (99.8) | 39.8 (12.1) | .96 | |  | | 841 (99.8) | | 39.9 (12.1) | | .98 | |  |
| Composite gait speed^a^, m/s | 904 (96.4) | 1.48 (0.19) |  | 872 (99.7) | 1.48 (0.19) | .97 | |  | | 840 (99.6) | | 1.48 (0.19) | | .69 | |  |
| 2-min step test | 886 (94.5) | 115.5 (26.6) |  | 855 (97.7) | 115.8 (26.4) | .76 | |  | | 825 (97.9) | | 116.1 (26.4) | | .56 | |  |
| Chair stands | 873 (93.1) | 18.3 (5.6) |  | 844 (96.5) | 18.4 (5.6) | .75 | |  | | 815 (96.7) | | 18.4 (5.6) | | .60 | |  |
| **Cognitive function** |  |  |  |  |  |  | |  | |  | |  | |  | |  |
| Adulthood IQ | 918 (97.9) | 99.4 (15.0) |  | 872 (99.7) | 99.6 (15.1) | .78 | |  | | 840 (99.6) | | 99.8 (14.9) | | .49 | |  |

Notes: BrainAGE = brain Age Gap Estimate; SD = standard deviation; suPAR = soluble urokinase plasminogen activator receptor.

^a^Gait speed was measured as an average across the three individual walk conditions (usual, dual task, and maximum gait speed) to generate the measure of composite gait speed.

| **eTable 3. Correlation coefficients for log-transformed C-reactive protein, ln(CRP), with cohort characteristics for participants in the Dunedin Study at age 45 years** | | | |
| --- | --- | --- | --- |
| **Variable** | **N** | ***r* (95% CI)** | ***P***^a^ |
| Total N | 873 |  |  |
| Male | 442 | -0.07 (-0.14 to -0.004)^b^ | .036 |
| **Health habits** |  |  |  |
| Current smoking | 871 | 0.06 (-0.005 to 0.13)^b^ | .07 |
| Physical activity | 873 | -0.14 (-0.20 to -0.07)^c^ | <.0001 |
| Alcohol use (drinks/week) | 870 | 0.02 (-0.05 to 0.09)^c^ | .56 |
| **Health** |  |  |  |
| Body mass index (kg/m^2^) | 873 | 0.47 (0.42 to 0.52)^c^ | <.0001 |
| Anti-inflammatory medication | 873 | 0.10 (0.04 to 0.17)^b^ | .0027 |
| Self-reported health | 872 | -0.20 (-0.27 to -0.14)^b^ | <.0001 |
| Current health conditions | 872 | 0.25 (0.19 to 0.32)^c^ | <.0001 |
| **Aging** |  |  |  |
| Pace of Aging^d^ | 872 | 0.48 (0.43 to 0.53)^c^ | <.0001 |
| Facial Age | 870 | 0.18 (0.11 to 0.24)^c^ | <.0001 |
| BrainAGE | 839 | 0.12 (0.05 to 0.18)^c^ | .0007 |
| **Functional capacity** |  |  |  |
| Physical limitations | 869 | 0.18 (0.12 to 0.25)^c^ | <.0001 |
| One-legged balance | 864 | -0.25 (-0.31 to -0.18)^c^ | <.0001 |
| Handgrip strength | 871 | -0.14 (-0.21 to -0.08)^c^ | <.0001 |
| Composite gait speed^e^ | 870 | -0.21 (-0.27 to -0.14)^c^ | <.0001 |
| 2-min step test | 853 | -0.19 (-0.25 to -0.12)^c^ | <.0001 |
| Chair stands | 842 | -0.24 (-0.30 to -0.17)^c^ | <.0001 |
| **Cognitive function** |  |  |  |
| Adulthood IQ | 870 | -0.16 (-0.22 to -0.09)^c^ | <.0001 |

*Notes:* BrainAGE = brain Age Gap Estimate; CI = confidence interval.

^a^Bonferroni-corrected P level = 0.003.

^b^Spearman correlation coefficient.

^c^Pearson correlation coefficient.

^d^CRP is part of the Pace of Aging measure.

^e^Gait speed was measured as an average across the three individual walk conditions (usual, dual task, and maximum gait speed) to generate the measure of composite gait speed.

| **eTable 4. Associations of age 45 measures of aging, functional capacity, and cognitive function with serum CRP (log-transformed) or suPAR levels at age 45 in n=875 participants in the Dunedin Study**^a^ | | | | | | | |
| --- | --- | --- | --- | --- | --- | --- | --- |
|  | **ln(CRP)** | | |  | **suPAR** | | |
| **Variable** | **N** | **β (95% CI)**^a^ | **P**^b^ |  | **N** | **β (95% CI)**^a^ | **P**^b^ |
| **Adjusted for sex** |  |  |  |  |  |  |  |
| **Aging** |  |  |  |  |  |  |  |
| Pace of Aging^c^ | 872 | 0.49 (0.43; 0.54) | <.0001 |  | 874 | 0.38 (0.32; 0.44) | <.0001 |
| Facial Age | 870 | 0.18 (0.12; 0.25) | <.0001 |  | 872 | 0.28 (0.22; 0.34) | <.0001 |
| BrainAGE | 839 | 0.10 (0.03; 0.17) | .0029 |  | 841 | 0.13 (0.06; 0.20) | .0001 |
| **Functional capacity** |  |  |  |  |  |  |  |
| Physical limitations | 869 | 0.18 (0.11; 0.24) | <.0001 |  | 871 | 0.32 (0.25; 0.38) | <.0001 |
| One-legged balance | 864 | -0.24 (-0.30; -0.18) | <.0001 |  | 866 | -0.20 (-0.26; -0.13) | <.0001 |
| Handgrip strength | 871 | -0.13 (-0.24; -0.02) | .023 |  | 873 | -0.19 (-0.30; -0.09) | .0004 |
| Composite gait speed^d^ | 870 | -0.20 (-0.26; -0.13) | <.0001 |  | 872 | -0.22 (-0.29; -0.16) | <.0001 |
| 2-min step test | 853 | -0.19 (-0.26; -0.12) | <.0001 |  | 855 | -0.18 (-0.22; -0.10) | <.0001 |
| Chair stands | 842 | -0.23 (-0.29; -0.16) | <.0001 |  | 844 | -0.21 (-0.25; -0.13) | <.0001 |
| **Cognitive function** |  |  |  |  |  |  |  |
| Adult IQ | 870 | -0.16 (-0.22; -0.09) | <.0001 |  | 872 | -0.24 (-0.31; -0.18) | <.0001 |
| Child-to-adult cognitive decline | 859 | -0.05 (-0.12; 0.01) | .12 |  | 861 | -0.14 (-0.21; -0.07) | <.0001 |
| **Adjusted for sex, BMI, and smoking** | | | | | | | |
| **Aging** |  |  |  |  |  |  |  |
| Pace of Aging^c^ | 869 | 0.29 (0.21; 0.37) | <.0001 |  | 871 | 0.41 (0.33; 0.50) | <.0001 |
| Facial Age | 868 | 0.10 (0.04; 0.16) | .0020 |  | 870 | 0.17 (0.11; 0.24) | <.0001 |
| BrainAGE | 838 | 0.05 (-0.01; 0.11) | .08 |  | 840 | 0.07 (0.01; 0.14) | .029 |
| **Functional capacity** |  |  |  |  |  |  |  |
| Physical limitations | 867 | 0.02 (-0.04; 0.09) | .42 |  | 869 | 0.24 (0.18; 0.31) | <.0001 |
| One-legged balance | 862 | -0.09 (-0.15; -0.03) | .0029 |  | 864 | -0.12 (-0.18; -0.05) | .0005 |
| Handgrip strength | 869 | -0.13 (-0.22; -0.03) | .0078 |  | 871 | -0.19 (-0.29; -0.09) | .0003 |
| Composite gait speed^d^ | 868 | -0.04 (-0.10; 0.02) | .23 |  | 870 | -0.14 (-0.21; -0.08) | <.0001 |
| 2-min step test | 852 | -0.08 (-0.14; -0.02) | .0076 |  | 852 | -0.08 (-0.13; -0.02) | .013 |
| Chair stands | 841 | -0.11 (-0.17; -0.05) | .0002 |  | 843 | -0.14 (-0.19; -0.07) | <.0001 |
| **Cognitive function** |  |  |  |  |  |  |  |
| Adult IQ | 868 | -0.08 (-0.14; -0.02) | .014 |  | 870 | -0.15 (-0.21; -0.08) | <.0001 |
| Child-to-adult cognitive decline | 857 | -0.03 (-0.09; 0.03) | .36 |  | 859 | -0.08 (-0.14; -0.01) | .017 |

*Notes:* BrainAGE = brain Age Gap Estimate; BMI = body mass index; CI = confidence interval; CRP = C-reactive protein; suPAR = soluble urokinase plasminogen activator receptor.

^a^Standardized β coefficients.

^b^Bonferroni-corrected P level = 0.005.

^c^CRP and BMI are part of the Pace of Aging measure.

^d^Gait speed was measured as an average across the three individual walk conditions (usual, dual task, and maximum gait speed) to generate the measure of composite gait speed.

| **eTable 5. Associations between age-45 measures of aging, functional capacity, and cognitive function and high suPAR levels.** | | | |
| --- | --- | --- | --- |
|  | **N** | **OR (95% CI)**^a^ | ***P*** |
| **Aging** |  |  |  |
| Pace of Aging^c^ | 874 | 2.23 (1.87-2.66) | <.0001 |
| Facial Age | 872 | 1.89 (1.58-2.27) | <.0001 |
| BrainAGE | 841 | 1.26 (1.06-1.49) | .0083 |
| **Functional capacity** |  |  |  |
| Physical limitations | 871 | 1.70 (1.46-1.98) | <.0001 |
| One-legged balance | 866 | 0.65 (0.54-0.78) | <.0001 |
| Handgrip strength | 873 | 0.79 (0.59-1.05) | .11 |
| Composite gait speed^d^ | 872 | 0.63 (0.53-0.76) | <.0001 |
| 2-min step test | 855 | 0.57 (0.46-0.70) | <.0001 |
| Chair stands | 844 | 0.54 (0.43-0.67) | <.0001 |
| **Cognitive function** |  |  |  |
| Adult IQ | 872 | 0.56 (0.46-0.67) | <.0001 |
| Child-to-adult cognitive decline | 861 | 0.77 (0.64-0.91) | .0023 |

Notes: BrainAGE = brain Age Gap Estimate; CI = confidence interval; OR = odds ratio; suPAR = soluble urokinase plasminogen activator receptor.
High suPAR levels are defined as >3.53 ng/mL, which corresponds to the highest suPAR quintile. We report sex-adjusted odds ratios predicting which participants had elevated suPAR (highest suPAR quintile). The ORs indicate that for every 1 SD unit increase in the predictor variable, the likelihood of having elevated suPAR increases by X times.

**eFigure 1. Accelerated Aging is Associated with Elevated suPAR at Age 45 Years.**

Generalized additive models of Pace of Aging, Facial Age, and brainAGE by suPAR concentration at age 45. Models were fitted with df=6, using the gam() package in R. suPAR, soluble urokinase plasminogen activator receptor.

**
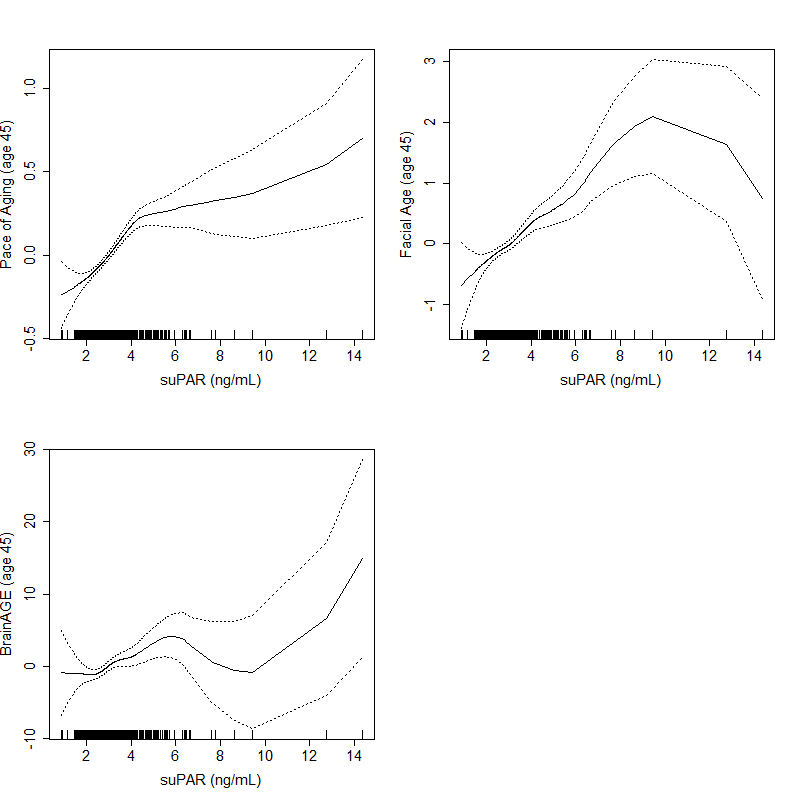
**

**eFigure 2. suPAR Levels by Alcohol Use (n=838).**

Participants were categorized as those drank within recommendations at both age 38 and age 45; those who drank more than recommended levels at age 38, but within recommendations at age 45; those who drank within recommendations at age 38, but more than recommended levels at age 45; and those who drank more than recommended levels at both age 38 and age 45. Panel **A** shows scatterplots of suPAR levels at age 38 and 45 years with black dots indicating mean suPAR. Panel **B** shows mean suPAR at age 38 and 45 years by alcohol use according to national recommendations with error bars indicating standard error. suPAR, soluble urokinase plasminogen activator receptor.


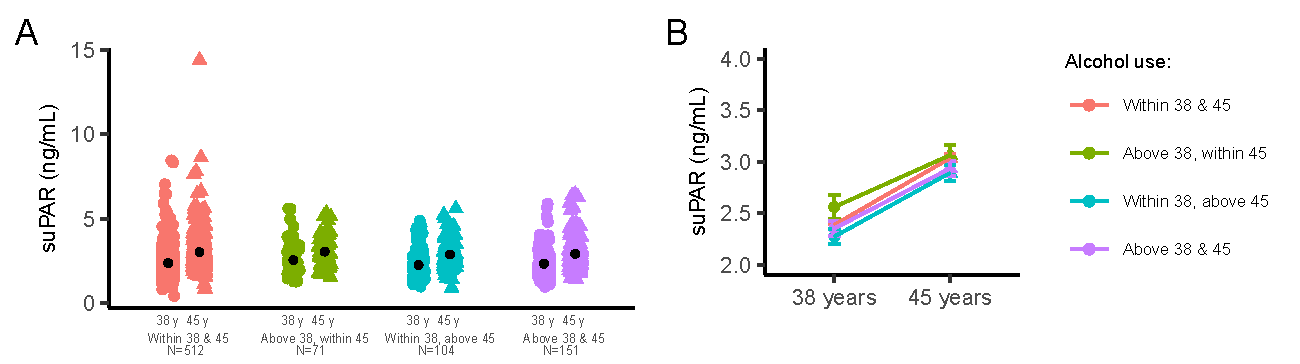


**REFERENCES**

1. Belsky DW, Caspi A, Houts R, et al. Quantification of biological aging in young adults. *Proc Natl Acad Sci U S A*. 2015;112(30):E4104-E4110. doi:10.1073/pnas.1506264112.

2. Cullinane EM, Siconolfi S, Carleton RA, Thompson PD. Modification of the Astrand-Rhyming sub-maximal bicycle test for estimating VO2max of inactive men and women. *Med Sci Sports Exerc*. 1988;20(3):317-318.

3. National Kidney Foundation. CKD-EPI CREATININE EQUATION. https://www.kidney.org/content/ckd-epi-creatinine-equation-2009. Published 2009. Accessed March 5, 2020.

4. World Health Organization. *Oral Health Surveys: Basic Methods, 2nd Ed*. World Health Organization; 1977.

5. World Health Organization. *Oral Health Surveys: Basic Methods, 4th Ed*. World Health Organization; 1997. https://apps.who.int/iris/handle/10665/41905.

6. Glasser MF, Sotiropoulos SN, Wilson JA, et al. The minimal preprocessing pipelines for the Human Connectome Project. *Neuroimage*. 2013;80:105-124. doi:10.1016/j.neuroimage.2013.04.127.

7. Greve DN, Fischl B. Accurate and robust brain image alignment using boundary-based registration. *Neuroimage*. 2009;48(1):63-72. doi:10.1016/j.neuroimage.2009.06.060.

8. Robinson EC, Jbabdi S, Glasser MF, et al. MSM: a new flexible framework for Multimodal Surface Matching. *Neuroimage*. 2014;100:414-426. doi:10.1016/j.neuroimage.2014.05.069.

9. Liem F, Varoquaux G, Kynast J, et al. Predicting brain-age from multimodal imaging data captures cognitive impairment. *Neuroimage*. 2017;148:179-188. doi:10.1016/j.neuroimage.2016.11.005.

10. Elliott ML, Belsky DW, Knodt AR, et al. Brain-age in midlife is associated with accelerated biological aging and cognitive decline in a longitudinal birth-cohort. *Mol Psychiatry*. 2019. doi:10.1038/s41380-019-0626-7.
